# Validation of the psychometric properties of the scale of resilience to suicide attempts among adolescents in Mainland China

**DOI:** 10.3389/fpsyt.2025.1543425

**Published:** 2025-04-10

**Authors:** Li Zhu, Wenqian Cheng, Yaoyao Huang, Siying Xin, Wan Shu, Xiaoning Wang, Jinsheng Zhang, Mei Li, QunFang Miao

**Affiliations:** ^1^ School of Nursing, Hangzhou Normal University, Hangzhou, Zhejiang, China; ^2^ Clinical Medicine, Hangzhou Normal University, Hangzhou, Zhejiang, China; ^3^ School of Nursing, Naval Medical University, Shanghai, China; ^4^ Affiliated Mental Health Center, Zhejiang University School of Medicine, Hangzhou, Zhejiang, China; ^5^ Mental Health Education and Counseling Center, Hangzhou Normal University, Hangzhou, Zhejiang, China

**Keywords:** adolescent, resilience, suicide, validation, Chinese

## Abstract

**Background:**

Suicide resilience has garnered increasing attention from researchers due to its potential role in suicide prevention. In 2021, Sánchez-Teruel et al. developed a tool to assess the resilience levels of individuals with a history of suicide attempts. The Scale of Resilience to Suicide Attempts (SRSA) is composed of 18 items across three dimensions: internal protection, emotional stability, and external protection. While the scale has shown robust psychometric properties in Spanish-speaking populations, cultural differences call for a revalidation of its psychometric characteristics among suicide attempters in Mainland China.

**Objective:**

This study aims to translate and adapt the SRSA into Chinese, and to evaluate its psychometric properties in adolescents who have attempted suicide in Mainland China.

**Methods:**

Following Brislin’s translation model, a survey was conducted using purposive sampling on 393 adolescents who had attempted suicide at the Hangzhou Normal University Affiliated Hospital and the Affiliated Mental Health Center of Zhejiang University School of Medicine. An expert panel evaluated the content validity. The scale’s structural validity was assessed through exploratory factor analysis and confirmatory factor analysis, along with measurement invariance. Additionally, tests for convergent validity, discriminant validity, and criterion-related validity were conducted. Reliability was evaluated using Cronbach’s alpha coefficient, McDonald’s omega, test-retest reliability, and split-half reliability.

**Results:**

The Chinese version of the SRSA comprises three dimensions and 16 items. The item-level content validity index for all items ranged from 0.88 to 1.00, while the Scale-Level Content Validity Index was 0.97. The three common factors explained a cumulative variance of 59.339%. Confirmatory factor analysis demonstrated a good model fit. The Cronbach’s alpha coefficient for the entire scale was 0.908, and McDonald’s omega was 0.910, with individual dimension Cronbach’s alpha values ranging from 0.780 to 0.869 and McDonald’s omega ranging from 0.859 to 0.910.

**Conclusion:**

The Chinese version of the SRSA is a valid and reliable tool for assessing the resilience levels of adolescents who have attempted suicide in Mainland China.

## 1 Introduction

Suicide is a significant global public health challenge. According to data from the World Health Organization in 2019, more than 700,000 people die by suicide each year worldwide (1). In China, suicide is one of the leading causes of death, with nearly 1 million individuals seeking medical treatment for suicide attempts annually (2), and approximately half of those who attempt suicide do not receive hospital care (3). Among adolescents aged 15-19, suicide has become the fourth leading cause of death (4). Studies indicate that the prevalence of suicidal ideation among Chinese middle school students ranges from 6.76% to 32%, while the incidence of suicide attempts is between 1.5% and 9.7% (5). Despite evidence suggesting a decline in China’s overall suicide rate over the past decade, adolescent suicide rates remain disproportionately high—approximately 1.5 times the global average for this age group (6). Adolescence represents a critical developmental transition, characterized by significant biological, emotional, cognitive, psychological, and behavioral changes. (7) Suicide attempts are associated with a wide range of adverse outcomes, leading to both physical and psychological impairments. Moreover, they significantly increase the risk of repeated attempts, which may ultimately result in suicide (8).

Historically, research on adolescent suicide attempts has predominantly concentrated on identifying risk factors (9, 10), with relatively scant emphasis on protective factors that could mitigate the occurrence of such behaviors. While some adolescents die during their first suicide attempt, others are able to reduce the psychosocial impact of risk factors through enhanced resilience, resulting in a decreased risk of subsequent attempts. Given the observation that many individuals at high risk for suicide do not ultimately die by suicide, there has been increasing interest in understanding the role of resilience in suicide prevention (11). Studies indicates that psychological resilience may inhibit or diminish the direct impact of risk factors that would otherwise contribute to suicidal ideation (12, 13). Furthermore, individuals with low resilience are at heightened risk for lifetime suicidal behavior (14). Resilience is increasingly recognized as a critical protective factor against suicide. Resilience refers to the interaction between protective and risk factors following adverse situations (such as unemployment, the loss of a family member, or a suicide attempt), which can facilitate appropriate personal growth and ultimately lead to optimal adaptive outcomes. (15) Unlike the concepts of strengths, ego-resilience, and toughness, strengths refer to inherent personal traits or abilities (16). Ego-resilience is a personality trait that emphasizes an individual’s flexibility, adaptability, and ability to recover when facing internal and external stressors in daily life, representing resilience at the intrapersonal level (17). Toughness emphasizes endurance and perseverance in the face of challenges (18). Resilience, however, is distinct in that it focuses on the outcome of recovery and adaptation following adversity, potentially leading to personal growth or transformation.

Most studies have utilized the Connor-Davidson Resilience Scale (CD-RISC), a tool developed by Connor et al. (19) to measure resilience in individuals with post-traumatic stress disorder symptoms. It has been applied across various age groups, genders, and clinical populations (20–23). However, there is limited research that employs specific tools to assess suicide resilience in populations with a history of suicidal ideation. Given the limitations of existing measurement instruments, in 2021, Sánchez-Teruel et al. developed the Scale of Resilience to Suicide Attempts (SRSA) to assess resilience in individuals with a history of suicide attempts. The scale comprises three dimensions—internal protection, emotional stability, and external protection—encompassing a total of 18 items. It employs a 5-point Likert scale, with higher total scores reflecting greater psychological resilience following a suicide attempt (24). This scale was tested in Spain, with studies involving 628 adolescents with a history of self-harm or suicide attempts, 229 adult women seeking treatment for self-harm or suicidal behavior, and 147 adults with a history of suicide attempts (24–26). The SRSA demonstrated excellent internal consistency, with a Cronbach’s α coefficient of 0.93 (25).This study aims to investigate the reliability and validity of the Chinese version of the SRSA, with the goal of providing a tool that can be used to assess adolescent suicide resilience levels in Mainland China.

## 2 Methods

### 2.1 Participants

This study employed a convenience sampling method to recruit adolescents who had attempted suicide and were receiving treatment at Hangzhou Normal University Affiliated Hospital and the Affiliated Mental Health Center, Zhejiang University School of Medicine between February and August 2024.Inclusion criteria: (1) Participants were aged 10-19 years, consistent with the World Health Organization’s definition of adolescence; (2) A history of at least one suicide attempted, in line with the definition of a suicide attempt meeting the definition of a suicide attempted. Exclusion criteria: Participants with other severe physical illnesses or major psychiatric disorders were excluded.

The sample size was determined based on the general principles of factor analysis, which recommend a minimum of 5 to 10 times the number of scale items (27). SRSA has 18 items, considering a 10%-20% rate of invalid responses, the required sample size for exploratory factor analysis (EFA) is at least 108 participants. For confirmatory factor analysis (CFA), a minimum sample size of 200 is recommended (28). Therefore, the estimated total sample size should be at least 308 participants. In this study, a total of 393 valid questionnaires were collected across two rounds: the first round included 178 responses for EFA, and the second round included 215 responses for CFA.

A total of 406 questionnaires were distributed, with 393 valid responses collected, resulting in an effective response rate of 96.80%. The ages of the 393 adolescents with ranged from 11 to 19 years, with a mean age of 15.09 ± 1.99 years. Demographic information is presented in **Table 1**.

**Table 1:** General information of the study participants (n=393).

Project		n	%
Gender	Male	155	39.44
	Female	238	60.56
Residence	Urban	211	53.69
	Rural	182	46.31
Educational attainment	Elementary School	38	9.67
	Junior High School	198	50.38
	High School	157	39.95
School enrollment status	Enrolled	93	23.66
	On Hiatus	133	33.84
	Intermittent schooling	167	42.50
Primary caregiver	Parents	233	59.29
	Mother	94	23.92
	Father	20	5.09
	Grandparents	42	10.69
	Other (Extended Family)	4	1.01
Only child status	Yes	123	31.30
	No	270	68.70

Parents’ marital status	Married	307	78.12
	Divorced	71	18.07
	Separated	9	2.29
	Other (Widowed)	6	1.52
Family history of mental illness	Yes	50	12.72
	No	343	87.28
Family history of suicide	Yes	18	4.58
	No	375	95.42
Suicide Attempt Count	1	136	34.61
	2	88	22.39
	>3	169	43.00
The most recent suicide time	Three months ago	248	63.10
	Six months ago	65	16.54
	One year ago	80	20.36

### 2.2 Measures

#### 2.2.1 General information questionnaire

The questionnaire was developed by the researchers based on a review of domestic and international literature. It includes items on gender, residence, educational attainment, school enrollment status, primary caregiver, only child status, parents’ marital status, family history of mental illness, family history of suicide, frequency, the most recent suicide time.

#### 2.2.2 Chinese version of the CD-RISC-10

The scale was developed by Campbell-Sills et al. based on a five-dimensional, 25-item scale co-authored by the American psychologists Connor and Davidson (29). In 2016, Chinese researcher Zengjie Ye translated the CD-RISC-10 into Chinese. Validation studies confirmed that the scale maintained its original single-factor structure in a sample of nursing students, explaining 48.641% of the total variance, with a Cronbach’s α of 0.851. (30). Additionally, the scale was validated in parents of children diagnosed with cancer, where it also demonstrated a good model fit for the single-factor structure, explaining 49.602% of the total variance, with a Cronbach’s α of 0.877 (31). In this study, the Cronbach’s α for the Chinese version of the CD-RISC-10 was 0.912.

#### 2.2.3 Chinese version of the SRSA

This scale consists of three dimensions: internal protection, emotional stability, and external environment. Each item is rated on a 5-point Likert scale, ranging from “never” (0 points) to “always” (4 points), with a total possible score of 0–64. Higher scores indicate a higher level of resilience in adolescents who have attempted suicide.

### 2.3 Psychometric testing procedures

This study initially contacted Professor Teruel, the original developer of the SRSA scale, via email to explain the background, purpose, and significance of the study, and to request permission for the translation and cultural adaptation of the SRSA scale. After obtaining formal authorization and consent, the original SRSA scale was acquired. The translation and adaptation process followed the guidelines for cross-cultural adaptation recommended by the International Test Commission (32), which includes five stages: translation, synthesis, back-translation, expert panel review, and pretesting.

#### 2.3.1 Stage 1: forward translation

Two bilingual translators, both native Chinese speakers with excellent proficiency in both languages, were invited to independently translate the scale. One translator was a PhD in nursing psychology with overseas study experience, and the other was a Master’s graduate in professional translation, with an International English Language Testing System (IELTS) score of 6.5. The translations produced versions A1 and A2.

#### 2.3.2 Stage 2: expert review and consensus

A psychiatric expert (Associate Chief Physician with 30 years of experience) independently reviewed the A1 and A2 versions. In consultation with the research team and the two translators, a consensus was reached, and a unified version, SRSA Chinese version A, was created.

#### 2.3.3 Stage 3: back-translation

Two additional translators, who had not seen the original scale, were recruited for the back translation process. One was a PhD nursing student with English proficiency (CET-6), and the other was a medical English instructor. They independently translated the SRSA Chinese version A back into English, resulting in back-translation versions B1 and B2. The researchers then conducted a comparative analysis of the differences between the back-translations and made necessary adjustments, ultimately developing the preliminary SRSA Chinese version B.

#### 2.3.4 Stage 4: expert panel review

A panel of eight experts was invited to review the translated scale. The panel included one Chief Physician in Clinical Psychology, two Associate Chief Physicians in Clinical Psychology, two Head Nurses from psychiatric hospitals specializing in adolescent psychological issues, one Associate Professor in Nursing Psychology, one expert in suicide crisis intervention, and one psychotherapist. The panel members held the following qualifications: three with doctoral degrees, four with master’s degrees, and one with a bachelor’s degree; three with senior titles and five with associate senior titles. Their collective experience and qualifications ensured a comprehensive evaluation of the scale. The panel’s feedback and suggested revisions included the following: (1) In item 11, the phrase “think about it” was considered insufficient to convey the depth of reflection, and the suggestion was made to replace it with “I think twice before acting,” which better aligns with the Chinese cultural context of emphasizing caution. (2) In item 17, the word “mock” was considered pejorative in Chinese, so the revision suggested was “I can laugh at problems and not let them get to me.” (3) In item 18, the word “always” was deemed too absolute, and it was suggested to replace it with “tend to,” which more accurately reflects habitual behavior and sounds more natural. The final version of the scale, SRSA Chinese version C, was developed after incorporating these revisions.

#### 2.3.5 Stage 5: pretesting

A pretest was conducted with 40 adolescents who had a history of suicide attempts, using the SRSA Chinese version C to assess the clarity and comprehensibility of each item. The results showed that the majority of adolescents found the questionnaire items to be clear and easy to understand, and they were able to complete the entire questionnaire in approximately 3 to 4 minutes, which was within an acceptable time frame. However, three adolescents reported difficulty understanding item 8: “I am as good as my peers/friends.” They felt that they did not fully recognize their strengths or advantages and found the phrasing somewhat abstract. Therefore, this item was revised to “In certain areas, I am as good as my peers/friends” to improve clarity. The final version of the SRSA Chinese test form was thus established.

### 2.4 Data collection

Data were collected using a combination of online and offline methods. All surveys were conducted anonymously, and the introductory section of the questionnaire clearly explained the study’s objectives, significance, and instructions for completing the survey. Participants were informed that they had the right to withdraw from the study at any time if they felt uncomfortable during the survey process. For the online survey, participants were able to complete the scale by scanning a QR code or clicking on a link. Considering that some hospitalized patients may have limited access to mobile devices, paper-based questionnaires were also provided. These were distributed on-site by the researchers, who also offered guidance on how to complete the forms.

All participants were fully informed about the study’s content and voluntarily consented to participate before data collection. During the data collection process, strict confidentiality protocols were followed to ensure the protection of all participants’ personal information.

### 2.5 Data analysis

The collected valid data were cross-checked by two researchers, then downloaded into Microsoft Excel for organization. Statistical analyses were conducted using IBM SPSS Statistics (version 25.0) and Mplus (version 8.3). The item analysis was conducted using the Critical Ratio and Correlation Coefficient methods. Validity testing included several approaches. Content validity was assessed through expert evaluation, with an Item-Level Content Validity Index (I-CVI) greater than 0.78 and a Scale-Level Content Validity Index (S-CVI) exceeding 0.90 considered acceptable. Structural validity was examined using both EFA and CFA. The results of EFA, conducted on Sample A (n_1_ = 178), revealed several latent factors with eigenvalues greater than 1 and a cumulative variance explained exceeding 50%, indicating robust structural validity. CFA was performed on Sample B (n_2_ = 215) using the robust maximum likelihood estimation method in Mplus software. The model fit was evaluated based on the criteria: χ²/df < 3.000, RMSEA < 0.050, SRMR<0.05 and CFI and TLI exceeding 0.900. Measurement invariance was assessed through multi-group CFA, evaluating configural, metric, and scalar invariance, with model comparisons based on ΔCFI < 0.01 and ΔRMSEA < 0.015. Convergent validity was supported by factor loadings exceeding 0.5, an Average Variance Extracted (AVE) above 0.4, and Composite Reliability (CR) greater than 0.8. Discriminant validity was verified using the Fornell-Larcker criterion, ensuring that the square root of the AVE exceeded the correlation coefficients between dimensions. Criterion-related validity was examined through Pearson correlation analysis between the SRSA scale and the Chinese version of the Resilience Scale, with higher correlation coefficients indicating stronger criterion validity. In reliability testing, if both Cronbach’s alpha and McDonald’s omega coefficients exceed 0.7, the measure demonstrates good internal consistency. Split-half reliability, assessed using the Spearman-Brown coefficient, is also considered acceptable if it surpasses 0.7, further confirming internal consistency. Additionally, test-retest reliability was evaluated with 40 highly cooperative participants, yielding a Pearson correlation coefficient greater than 0.7 between the two administrations, indicating strong temporal stability (33). The significance level for all tests was set at α = 0.05.

### 2.6 Ethics considerations

This study was approved by the Ethics Committees of Affiliated Mental Health Center, Zhejiang University School of Medicine (2023058) and The Affiliated Hospital of Hangzhou Normal University (2024-E2-KS-149) During the study, the researchers adhered to the principles of informed consent, ensuring that all participants voluntarily agreed to participate and signed consent forms before data collection. Additionally, the study followed strict confidentiality protocols. All survey results were used solely for academic research purposes, and personal information was anonymized by using numerical codes in place of identifying details during data entry to ensure participant privacy.

## 3 Results

### 3.1 Item analysis

#### 3.1.1 Critical ratio method

The project analysis results utilize the critical ratio method and correlation coefficients. Each item’s total score is ranked from highest to lowest. The high group consists of the top 27% of the total scores (total score ≤ 35), while the low group comprises the bottom 27% of the total scores (total score ≥ 47). The results indicated that Items 1 and 17 had t-values of 2.834 (P = 0.005) and 2.138 (P = 0.034), respectively. All other items had t-values greater than 3.000 (t=10.794~17.814 P < 0.001).

#### 3.1.2 Correlation coefficient method

Pearson correlation analysis was conducted to assess the relationship between each item and the total score of the questionnaire. The analysis revealed that the correlation coefficients between the scores of Items 1 and Item 17 and the total score were 0.178 (P < 0.05) and 0.142 (P < 0.05), respectively. The correlation coefficients for the remaining items ranged from 0.561 to 0.775 (P < 0.05). Based on both the critical ratio method and the correlation coefficient method, Items 1 and 17 were found to perform poorly and were therefore removed.

### 3.2 Content validity analysis

Eight experts participated in the evaluation of the scale’s content validity, utilizing a four-point Likert scale where 1 denotes ‘irrelevant,’ 2 ‘weakly relevant,’ 3 ‘moderately relevant,’ and 4 ‘highly relevant.’ To thoroughly assess the content validity of the scale, both the I-CVI and S-CVI were computed. The findings revealed that I-CVI values ranged between 0.88 and 1.00, and the overall S-CVI was 0.97, both of which satisfy established validity criteria.

### 3.3 Structural validity analysis

#### 3.3.1 Exploratory factor analysis

EFA conducted on sample A (n=178) revealed a Kaiser-Meyer-Olkin (KMO) value of 0.900, and Bartlett’s test of sphericity was significant (χ² = 1287.143, P < 0.001), indicating that the data were suitable for factor analysis and that the common factor extraction method could be used to explain the majority of the variance in the questionnaire items. Principal component analysis with varimax rotation yielded three factors with eigenvalues greater than 1. Factor loadings for each item exceeded 0.4, with each factor’s loading being higher than those of other factors. The factor loadings ranged from 0.593 to 0.779, with the lowest being 0.593 and the highest being 0.779. Item 13 was associated with two factors with eigenvalues greater than 4, where Factor 2 had a loading of 0.593, and Factor 3 had a loading of 0.434, suggesting a stronger correlation with Factor 2. After discussion by the research team, Item 13 was retained. The cumulative variance explained by the three common factors was 59.835%, which is within an acceptable range. Detailed results are presented in **Table 2**.

**Table 2:** The factor loading matrix table of the Chinese version SRSA test manuscript (n_1_ = 178).

Iten	Factor loading			Communality (common factor variance)
	Factor1	Factor2	Factor3	
Item 2	0.282	0.239	**0.795**	0.769
Item 5	0.241	0.218	**0.611**	0.479
Item 8	0.187	0.151	**0.662**	0.496
Item 18	0.040	0.291	**0.657**	0.518
Item 4	0.336	**0.722**	0.398	0.793
Item 6	0.148	**0.608**	0.359	0.521
Item 9	0.063	**0.779**	0.088	0.618
Item 10	0.152	**0.735**	0.160	0.589
Item 11	0.237	**0.598**	0.192	0.451
Item 16	0.258	**0.685**	0.213	0.581
Item 3	**0.802**	0.194	0.237	0.737
Item 7	**0.777**	0.179	-0.011	0.635
Item 12	**0.636**	0.189	0.215	0.486
Item 13	**0.625**	0.131	**0.425**	0.588
Item 14	**0.746**	0.146	0.107	0.589
Item 15	**0.723**	0.211	0.276	0.643

Items in bold black indicate factor loadings greater than 0.4.

#### 3.3.2 Confirmatory factor analysis

**Figure 1:**
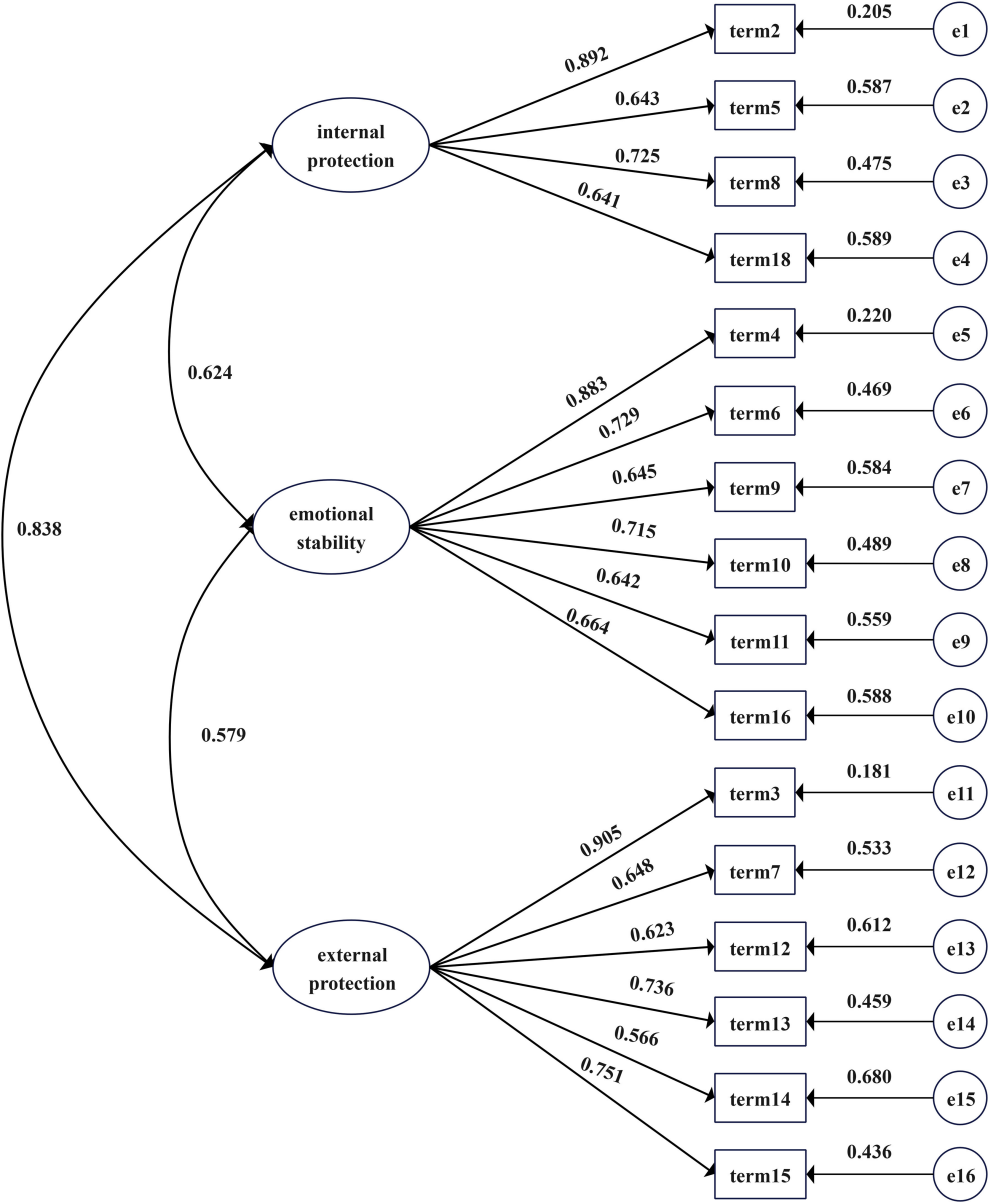
CFA model diagram.

EFA revealed three common factors, corresponding to the three dimensions specified in the original questionnaire: internal protection, emotional stability, and external protection. These were conceptualized as latent variables. The 16 items from the questionnaire were designated as observed variables to construct the model for CFA. The data from sample B did not meet the assumption of multivariate normality (Mardia statistic 485.2), Therefore, a robust maximum likelihood method was used for the analysis. The model’s fit was evaluated using data from sample B, demonstrating a robust fit, with a χ²/df ratio of 1.174, an RMSEA of 0.028, and a CFI of 0.985. The specific values are shown in **Table 3**, and the structural equation model for the CFA of the Chinese version of the SRSA test is presented in **Figure 1**.

**Table 3:** Fit indices of the model (n_2_ = 215).

Statistical test statistic	Result values	Critical values for fit
χ^2^/df	1.174	<3.000
RMSEA	0.028	<0.050
CFI	0.987	>0.900
TLI	0.985	>0.900
SRMR	0.039	<0.05

#### 3.3.3 Measurement invariance

Measurement invariance was assessed across gender, residence, educational attainment, and school enrollment status, which were treated as categorical variables. In the configural invariance model (M0), factor loadings and variances were freely estimated for groups delineated by gender, residence, educational attainment, and school enrollment status. Fit indices for these groups were reported as follows: for gender, χ²(202) = 250.764, CFI = 0.971, RMSEA = 0.047 [95% CI: 0.024, 0.065]; for residence, χ²(202) = 215.327, CFI = 0.992, RMSEA = 0.025 [95% CI: 0.000, 0.049]; for educational attainment, χ²(329) = 424.165, CFI = 0.930, RMSEA = 0.075 [95% CI: 0.057, 0.091]; and for school enrollment status, χ²(303) = 407.280, CFI = 0.936, RMSEA = 0.090 [95% CI: 0.051, 0.086]. Following confirmation of the configural invariance model, the equivalence of factor loadings across groups was subsequently tested. Comparisons of fit indices between the metric invariance model (M1) and the configural model (M0) showed no significant changes (ΔCFI ≤ 0.010, ΔRMSEA ≤ 0.015). Item intercepts were then constrained to equality across groups, with analyses between the scalar invariance model (M2) and the metric invariance model (M1) also indicating no significant differences (ΔCFI ≤ 0.010, ΔRMSEA ≤ 0.015). Therefore, the factor structure, factor loadings, and item intercepts of the SRSA scale demonstrated equivalence across gender, residence, educational attainment, and school enrollment status in adolescents. Detailed results are presented in **Table 4**.

**Table 4:** Measurement invariance (n_2_ = 215).

Model	χ^2^	*df*	CFI	RMSEA [90%CI]	△CFI	△RMSEA
Gender						
M0 (configural)	250.764	202	0.971	0.047 [0.024,0.065]	–	–
M1 (metric)	263.112	215	0.971	0.046 [0.022,0.064]	0.000	0.001
M2 (scalar)	272.577	228	0.973	0.043 [0.017,0.061]	0.002	0.003
Residence						
M0 (configural)	215.327	202	0.992	0.025 [0.000,0.049]	–	–
M1 (metric)	236.676	215	0.987	0.031 [0.000,0.052]	0.005	0.006
M2 (scalar)	247.213	228	0.988	0.028 [0.000,0.050]	0.001	0.003
Educational attainment						
M0 (configural)	424.165	303	0.930	0.075 [0.057,0.091]	–	–
M1 (metric)	449.926	329	0.930	0.072 [0.054,0.088]	0.000	0.003
M2 (scalar)	466.303	355	0.935	0.066 [0.048,0.082]	0.005	0.006
School enrollment status						
M0 (configural)	407.28	303	0.938	0.069 [0.051,0.086]	–	–
M1 (metric)	444.127	329	0.932	0.070 [0.052,0.086]	0.006	0.001
M2 (scalar)	464.846	355	0.935	0.066 [0.048,0.082]	0.003	0.004

#### 3.3.4 Convergent validity

The AVE for the three dimensions of the Chinese version of the SRSA test ranged from 0.515 to 0.536, exceeding the recommended threshold of 0.5. This demonstrates that the measurement items for each dimension possess strong convergent validity. Furthermore, the CR values, ranging from 0.819 to 0.863 and surpassing. The acceptable threshold of 0.7, indicate satisfactory internal consistency across the dimensions. The detailed values are shown in **Table 5**.

**Table 5:** Standardized regression coefficients for each item in the model (n_2_ = 215).

Dimension	Average Variance Extracted (AVE)	Composite Reliability (CR)
Internal Protection	0.536	0.819
Emotional Stability	0.515	0.863
External Protection	0.517	0.862

#### 3.3.5 Discriminant validity

The three dimensions of the Chinese version of the SRSA test demonstrated significant intercorrelations, with coefficients ranging from 0.5 to 0.7 (P < 0.05). Moreover, the square roots of the AVE for each dimension exceeded the respective correlation coefficients between the dimensions, confirming that the dimensions are distinctly differentiated and exhibit strong discriminant validity. Details are provided in **Table 6**.

**Table 6:** Analysis of discriminant validity results (n_2_ = 215).

	Internal Protection	Emotional Stability	External Protection
Internal Protection	0.732		
Emotional Stability	0.517***	0.718	
External Protection	0.737***	0.507***	0.719
AVE	0.856	0.847	0.848

*** p<0.05.

#### 3.3.6 Criterion-related validity

Compared to the Chinese version of the CD-RISC-10, the correlation coefficients for the dimensions ranged from 0.516 to 0.663 (P < 0.05), and the total score showed a correlation coefficient of 0.661 (P < 0.05), all exceeding 0.5. This indicates that the SRSA scale has good criterion-related validity. The detailed values are provided in **Table 7**.

**Table 7:** Criterion validity (n_2_ = 215).

Dimension	Internal Protection	Emotional Stability	External Protection	Total Score
Total Score - Criterion	0.653**	0.528**	0.528**	0.650**

**p<0.01.

### 3.4 Reliability analysis

The reliability test results of the Chinese version of the SRSA test show that the overall Cronbach’s α coefficient was 0.908, with the Cronbach’s α coefficients for the individual dimensions ranging from 0.778 to 0.869. The McDonald’s ω coefficient for the overall scale was 0.910, with values for the individual dimensions ranging from 0.793 to 0.863, further supporting the internal consistency of the scale. The split-half reliability coefficient was 0.845, with the split-half reliability coefficients for the dimensions ranging from 0.778 to 0.873. The test-retest reliability was 0.893, with the test-retest reliabilities for the individual dimensions ranging from 0.776 to 0.790. These results indicate that the questionnaire demonstrates good reliability. Detailed values can be found in **Table 8**.

**Table 8:** Reliability test results.

	Cronbach’s α	McDonald’s w	Split-half Reliability	Test-Retest Reliability (n=40)
Internal Protection	0.780	0.793	0.778	0.766
Emotional Stability	0.853	0.859	0.814	0.776
External Protection	0.869	0.863	0.873	0.790
Total Scale	0.908	0.910	0.841	0.893

## 4 Discussion

The aim of this study was to validate the SRSA scale for use in mainland China. The results indicated that, compared to the original scale, items 1 and 17 exhibited lower item discrimination and correlation, leading to their exclusion. After removing these two items, the scale achieved ideal levels of reliability and validity.

The results of the EFA showed item 13 (“I share and discuss my problems with family or friends “) extracted two factors with eigenvalues greater than 4. The factor loading for this item in the “External Protection” dimension was 0.624, while its factor loading in the “Emotional Stability” dimension was 0.434, indicating a stronger relationship with the “External Protection” dimension. After discussion by the research team, it was determined that seeking support from family or friends reflects “External Protection.” This behavior indicates an individual’s tendency to rely on external resources for emotional support and practical assistance. Furthermore, expressing and sharing difficulties serves as an emotional management strategy, contributing to emotional stability, which may depend on external relational support. Therefore, item 13 was included in the “External Protection” dimension. CFA identified three primary factors within the Chinese version of the SRSA: Internal Protection, Emotional Stability, and External Protection. This scale and its dimensions demonstrated strong correlations with the General Resilience Scale (CD-RISC), exhibiting correlation coefficients (r) exceeding 0.5 (p < 0.05). Reliability analyses indicated high levels of internal consistency for the Chinese SRSA, as evidenced by Cronbach’s alpha and McDonald’s omega coefficients. These findings are in alignment with those reported by Sánchez-Teruel et al. for the Spanish population (24–26), and surpass the metrics found in the Suicide Resilience Inventory-25 (SRI-25) developed by Osman et al. (34). The observed discrepancies may stem from differences in the study populations; our research focused on individuals with a history of suicide attempts, whereas Osman et al. examined a non-psychiatric sample of youth and university students, primarily measuring suicide ideation. Considering the differing severity between suicide ideation and attempts, resilience assessment outcomes are likely to vary. Furthermore, when Peter et al. administered the SRI-25 to patients with mental disorders, the heterogeneity in suicide-related behaviors within the sample emerged as a limitation of the SRI (35).

Previous studies have indicated that suicidal behaviors among adolescents are often concealed, as they might not explicitly express suicidal thoughts or intentions (36). Subtle changes in behavior can be challenging for teachers or peers to identify. Moreover, adolescents who have attempted suicide frequently face personal stigma, characterized by feelings of shame, labeling, and discrimination (37).During psychometric evaluations, Teachman et al. observed that participants displayed neutral attitudes towards mental illness on explicit measures; however, implicit measures uncovered underlying negative perceptions (38). In the context of Chinese culture, where suicide and mental health issues are often stigmatized (39), the presence of suicide-related terms in a scale may hinder individuals with a history of suicide from fully disclosing their true attitudes towards suicide.

The Suicide Resilience Scale for Adolescents (SRSA) uniquely assesses resilience by focusing on protective factors that highlight individual strengths and resources, rather than weaknesses and risks. Significantly, it avoids using suicide-related words or phrases, which facilitates a more authentic capture of adolescents’ resilience, without centering on potential vulnerabilities and risks. This approach enables a more accurate assessment of resilience in this sensitive group.

Additionally, Sánchez-Teruel et al. discovered that lower scores on the SRSA could predict suicide reattempts within six months, underscoring the scale’s not only diagnostic but also prognostic significance in assessing resilience among adolescents with a history of suicide attempts (25). This highlights its crucial role in suicide prevention strategies. The current study has primarily concentrated on translating and culturally adapting the SRSA for the Chinese context. Future research should aim to further validate the predictive utility of the Chinese version of the SRSA and investigate its practical applications in suicide intervention and prevention strategies. This will enhance our understanding of its effectiveness in different cultural settings and contribute to more targeted and effective suicide prevention efforts.

## 5 Limitations

This study has several limitations. Firstly, this study was conducted with a sample limited to Zhejiang Province, China, which may affect the generalizability and external validity of the findings. Future research should expand to a broader geographic range to enhance the reliability and validity of the scale, as well as improve the external applicability of the results. Second, the number of male participants in the sample was significantly lower than that of female participants, which may lead to insufficient gender representation in the study results. Future research should focus on improving gender balance to enhance the scale’s applicability across different demographic groups. Furthermore, to accommodate the adolescent population, this study adjusted the language and number of items in the original scale, which may reflect differences in social interactions and cognitive development stages between adolescents and adults in mainland China. Therefore, if the scale is applied to an adult population, its applicability should be re-validated to ensure the accuracy and reliability of the measurements.

## Data Availability

The raw data supporting the conclusions of this article will be available from the corresponding author on reasonable request.
